# Acute Inflammation Induces Neuroendocrine and Opioid Receptor Genes Responses in the Seabass *Dicentrarchus labrax* Brain

**DOI:** 10.3390/biology11030364

**Published:** 2022-02-24

**Authors:** Rita Azeredo, Marina Machado, Patricia Pereiro, Andre Barany, Juan Miguel Mancera, Benjamín Costas

**Affiliations:** 1 Centro Interdisciplinar de Investigação Marinha e Ambiental (CIIMAR), Universidade do Porto, 4450-208 Matosinhos, Portugal; mcasimiro@ciimar.up.pt (M.M.); patriciapereiro@iim.csic.es (P.P.); 2 Instituto de Investigaciones Marinas (IIM-CSIC), 36208 Vigo, Spain; 3 Department of Biology, Faculty of Marine and Environmental Sciences, Instituto Universitario de Investigación Marina (INMAR), Campus de Excelencia Internacional del Mar (CEI·MAR), University of Cadiz, 11519 Puerto Real, Spain; andre.barany@uca.es (A.B.); juanmiguel.mancera@uca.es (J.M.M.); 4 Instituto de Ciências Biomédicas Abel Salazar (ICBAS-UP), Universidade do Porto, 4050-313 Porto, Portugal

**Keywords:** genomics, stress response, HPI-axis, neuroendocrine-immune interaction

## Abstract

**Simple Summary:**

It is generally accepted (in mammals and in teleost fish, too) that stressful conditions affect the performance of an immune response. What is still far from being known is at what extend does an immune process affects the neuroendocrine system. Vaccination for instance, is nowadays a common practice in aquaculture and little is known about its physiological implications other than immunization. Here is a first approach to the study of the European seabass’ brain gene expression patterns in response to a peripheral inflammatory process. Genes related to the stress response were focused, along with those related to the opioid system. Increased expression of certain genes suggests the activation of a stress response triggered by inflammatory signals. Additionally, contrasting expression patterns of the same gene (increased vs decreased) in the different brain regions (as well as the time needed for changes to happen) point at different functions. These results clearly show the reactivity of different brain responses to an immune response, highlighting the importance of further studies on downstream implications (behavior, feeding, welfare, reproduction).

**Abstract:**

In fish, as observed in mammals, any stressful event affects the immune system to a larger or shorter extent. The neuroendocrine-immune axis is a bi-directional network of mobile compounds and their receptors that are shared between both systems (neuroendocrine and immune) and that regulate their respective responses. However, how and to what extent immunity modulates the neuroendocrine system is not yet fully elucidated. This study was carried out to understand better central gene expression response patterns in a high-valued farmed fish species to an acute peripheral inflammation, focusing on genes related to the hypothalamus-pituitary-interrenal axis and the opioid system. European seabass, *Dicentrarchus labrax*, were intra-peritoneally injected with either Freund’s Incomplete Adjuvant to induce a local inflammatory response or Hanks Balances Salt Solution to serve as the control. An undisturbed group was also included to take into account the effects due to handling procedures. To evaluate the outcomes of an acute immune response, fish were sampled at 4, 24, 48, and 72 h post-injection. The brain was sampled and dissected for isolation of different regions: telencephalon, optic tectum, hypothalamus, and pituitary gland. The expression of several genes related to the neuroendocrine response was measured by real-time PCR. Data were statistically analyzed by ANOVA and discriminant analyses to obtain these genes’ responsiveness for the different brain regions. Serotonergic receptors were upregulated in the telencephalon, whereas the optic tectum inhibited these transcription genes. The hypothalamus showed a somewhat delayed response in which serotonin and glucocorticoid receptors were concerned. Still, the hypothalamic corticotropin-releasing hormone played an important role in differentiating fish undergoing an inflammatory response from those not under such conditions. Opioid receptors gene expression increased in both the hypothalamus and the telencephalon, while in the optic tectum, most were downregulated. However, no changes in the pituitary gland were observed. The different brain regions under immune stimulation demonstrated clear, distinct responses regarding gene transcription rates as well as the time period needed for the effect to occur. Further, more integrative studies are required to associate functions to the evaluated genes more safely and better understand the triggering mechanisms.

## 1. Introduction

Inadequate aquaculture rearing conditions (husbandry, transportation, crowding densities, water parameters, etc.) often result in stressful environments that compromise fish growth and welfare [1,2,3]. Although these are the most obvious and relevant outcomes for fish farmers, other physiological responses, such as fish immune defenses, can be similarly compromised. Indeed, the extension of stress effects on fish health has mainly been investigated. It is now generally acknowledged that chronic stressful rearing conditions jeopardize fish immune response since it downregulates several immune defense mechanisms [4]. A fish farm ultimately decreases fish immune resistance upon a disease outbreak, leading to high mortalities.

Neuroendocrine and immune responses are tightly connected in what is known as the neuroendocrine-immune axis that comprises both the brain and the head-kidney. Teleost fish head-kidney presents endocrine and immune tissues as well as a complex paracrine signaling network, acting as an intermediary organ between neuroendocrine and immune systems. Neuroendocrine-immune processes are bidirectional, and so not only does stress (internal or external) modulate the immune response, but immunological processes are also able to trigger the hypothalamus-pituitary-interrenal (HPI) axis [5].

The regulators and effectors of these modulatory mechanisms belong to several molecular classes—from neuropeptides, opioids, and neurotransmitters to interleukins and chemokines. Moreover, several of these players are shared by both systems (immune and neuroendocrine). A fair amount of studies has been devoted to evaluating stress-induced effects on fish immunity. Most of these studies used cortisol as the primary stress marker as well as plasma glucose and lactate as indicators of secondary stress responses [6]. Upstream, hypothalamic corticotropin-releasing hormone (*crh*) and corticotropin-releasing hormone binding protein (*crhbp*), as well as pituitary proopiomelanocortin (*pomc*) gene expression are also good primary stress markers [7]. Serotonin, a tryptophan metabolite that mostly acts as a neurotransmitter at central levels, also presents a role in the stress responses modulating corticotropin-releasing hormone (CRF) and adrenocorticotropin hormone (ACTH) secretion. Often, when stress-inducing factors are persistent, this neuroendocrine response becomes chronic and suppresses immune mediators’ function, both by a metabolic reorganization that reduced influx of energy and by a direct inhibitory effect of cortisol [4,8,9].

Although the bidirectional aspect of this neuroendocrine-immune axis is well-acknowledged, the effects that an immunological process have on the HPI axis are far more disregarded than those inflicted by stress on immunity. In fish, such mechanisms have been reviewed by Verburg-van Kemenade and co-workers [5] and in more detail by Engelsma and colleagues [10], where the role of cytokines is given particular emphasis, as well as the pathways through which an immune process communicates with the central nervous system. Nonetheless, the neuronal arm of the HPI axis has far more branches connecting to the central backbone of the CRH and ACTH [7]. In this way, the serotonergic system, for instance, not only operates mood and behaviour but it also regulates the stress response (and ultimately cortisol release) using a considerable network of serotonin receptors [11,12,13]. Specifically, these receptors present a widespread brain distribution; however, it is not homogenous throughout different brain regions both in terms of abundancy and of identity, suggesting distinct functions and reactivity [14].

Likewise, and though comparatively even less explored, opioid receptors display a ubiquitous central distribution that is linked to their diverse plethora of functions. Opioids have long been associated to mood, behaviour, and nociception in fish [15]. However, they are also involved in regulatory mechanisms of both the immune and the stress responses [16,17]. At least in what carp (*Cyprinus carpio*) is concerned, opioids effects in leucocytes have been shown to be evolutionarily conserved [18]. Nevertheless, their exact roles and responsiveness to immune stimulation in the brain are still not known.

In an attempt to further understand the extent of peripheral immune signaling impact on central neuroendocrine responses, the present study intends to unveil and characterize the central neuronal gene expression profile, focusing on neuroendocrine and opioid receptors, in response to an acute peripheral inflammation in a marine fish species, the European seabass (*Dicentrarchus labrax*).

## 2. Material and Methods

### 2.1. Fish and Experimental Design

European seabass juveniles (*n* = 72, 87.3 g ± 16.5 body mass) were acquired from a certificated hatchery (MARESA, Spain) and maintained at the facilities of *Servicios Centrales de Investigación en Cultivos Marinos* (SCI-CM, CASEM, University of Cadiz, Puerto Real, Cádiz, Spain; Facilities for Breeding, Supplying and Users of Experimental Animals; Spanish Operational Code REGA ES11028000312). The fish were acclimated for 2 weeks in a flow-through 2 m^3^-tank. They were then transferred to a flow-through seawater system composed of sixteen 80 L-fiber glass tanks and fed a commercial diet for 30 days. The fish were maintained under natural photoperiod (June–July 2017, 36°31′45″ N, 6°11′31″ W), temperature (18–19 °C), and salinity (39 g L^−1^). Supplemental aeration was provided to maintain dissolved oxygen at 6.8 ± 0.4 mg L^−1^. Ammonia (<0.1 mg L^−1^), nitrite (<0.2 mg L^−1^), and nitrate (<50 mg L^−1^) were determined once weekly. Fish were fed twice per day (9:00 a.m. and 1:00 p.m.) at a rate of 2% of their body weight over the 30-day feeding trial. At the end of this period, eight fish were netted and euthanized by anaesthetic overdose (1 mL of 2-phenoxyethanol l^−1^ seawater; Merck, Darmstadt, Germany). Brains were collected and dissected into telencephalon, optic tectum, hypothalamus, and pituitary gland. Samples were kept in RNAlater (Sigma) at 4 °C for 24 h and finally stored at −20 °C until further processing. This group of fish, sampled before any intervention, was subsequently designated as undisturbed fish (0 h). The remaining fish were anesthetized (0.5 mL of 2-phenoxyethanol l^−1^ seawater) and intraperitoneally (i.p.) injected with 100 μL of either Freund’s Incomplete Adjuvant (FIA) to induce inflammation [19], or Hanks Balanced Salt Solution (HBSS) to serve as a sham group (CTRL), and reallocated in duplicate tanks of the original system for each experimental condition (n = 8 per condition). The fish were then sampled at 4, 24, 48, and 72 h, as previously described, following i.p. injection (n = 4 per tank, n = 8 per time and experimental group). They were fasted for 24 h before sampling and i.p. injection as well as during the experimental time.

All the experimental procedures complied with the University of Cádiz (Spain) guidelines and the European Union Council (2010/63/EU) to use animals in research. The experimental procedures were previously approved by the Spanish Government’s Ethics and Animal Welfare Committee (RD53/2013) and endorsed by the Regional Government (Junta de Andalucía reference number 28-04-15-241). All animal protocols were performed under Group-D licenses accredited by FELASA (Federation of European Laboratory Animal Science Associations).

### 2.2. Gene Expression

Total RNA isolation was conducted with the NZY Total RNA Isolation kit (NZYTech, Lisbon, Portugal) following the manufacturer’s specifications. RNA was quantified using the DS-11 Spectrophotometer (DeNovix), and first-strand cDNA was synthesized with NZY First-Strand cDNA Synthesis Kit (NZYTech, Lisbon, Portugal). Quantitative PCR assays were performed with CFX384 Touch Real-Time PCR Detection System, using 4.4 μL of diluted cDNA mixed with 5 μL of NZYSpeedy qPCR Green Master Mix^®^ and 0.3 μL (10 μM) of each specific primer in a final volume of 10 μL. The cDNA amplification was carried out with specific primers for genes that have been selected for their involvement in the neuroendocrine response. Primers were designed with NCBI Primer Blast Tool and IDT OligoAnalyzer Tool^TM^, respecting known qPCR restrictions (amplicon size, Tm difference between primers, GC content, and self-dimer or crossdimer formation). Part of the template sequences were obtained from available data in NCBI, while others were identified after searching the databases dicLab v1.0c seabass genome [20] and designed as previously described. The efficiency of primer pairs was analysed in serial, 2-fold dilutions of cDNA by calculating the slope of the regression line of the cycle thresholds (Ct) vs. the relative concentration of cDNA. Accession number, efficiency values, annealing temperature, product length, and primers sequences are presented in Table 1. Melting curve analysis was also performed to verify that no primer dimers were amplified. The standard cycling conditions were 95 °C initial denaturation for 10 min, followed by 40 cycles of two steps (95 °C denaturation for 15 s followed by primer annealing temperature for 1 min), 95 °C for 1 min followed by 35 s at the annealing temperature, and finally, 95 °C for 15 s. All reactions were carried out as technical duplicates. The expression of the target genes was normalized using the geometric mean of European seabass ribosome 40s subunit (*40s*) and elongation factor 1α (*ef1α*) expression levels and calculated according to the Pfaffl method [21].

### 2.3. Statistical Analysis

Gene expression values are presented as mean ± standard deviation (mean ± SD). Data were analysed for normality and homogeneity of variance, and, when necessary, outliers were removed, and data were log-transformed before being treated statistically. Possible i.p. injection effects were detected by One-way ANOVA, while inflammation- and sampling time-induced effects were identified using a two-way ANOVA. When statistical significance was detected, ANOVA analyses were followed by Tukey post-hoc test to identify differences within experimental treatments. These statistical analyses were performed using the computer package Statistica 13 for Windows. The level of significance used was *p* ≤ 0.05 for all statistical tests. In an attempt to discriminate and characterize brain regions of fish under inflammatory conditions, a multivariate canonical discriminant analysis was performed on each brain region dataset. Thereby, numerous combinations of the original variables (discriminant functions) were evaluated. Each discriminant function explains part of the total variance of the dataset and is loaded by variables contributing the most to that variation. Wilk’s λ test assessed discriminatory effectiveness, and the distance between group centroids was measured by squared Mahalanobis distance. To attest whether these distances were statistically significant, Fisher’s F statistic was performed. Discriminant analyses were carried out using the data analysis tool XLSTAT for Microsoft Office Excel, and a significance level of 95% (*p* ≤ 0.05) was used.

## 3. Results

For clarity, the results are presented in two main subsections: (i) the first one gathers genes more directly related to the HPI-axis response (results from Section 3.1), and (ii) the second one looks separately at the opioid receptors response. Notwithstanding their involvement in the same neuroendocrine pathways, they are relatively poorly studied for their role during inflammation (results from Section 3.2). Moreover, within each subsection and given the amount of data collected, relevance will be granted to (i) the i.p. injection effect and (ii) inflammation-induced changes. Intraperitoneal injection per se (regardless of content nature) was considered to modulate neuroendocrine gene expression patterns when both groups simultaneously behaved significantly differently from undisturbed fish (0 h). Inflammation was thought to affect gene expression whenever (i) there were significant differences between CTRL and FIA groups or (ii) whenever there was a difference between 0 h and FIA fish, without CTRL being different from 0 h. Due the high amount of results obtained, the complete set of gene expression results is provided as a Supplementary File.

### 3.1. HPI-Axis Response

#### 3.1.1. Telencephalon

**Figure 1:**
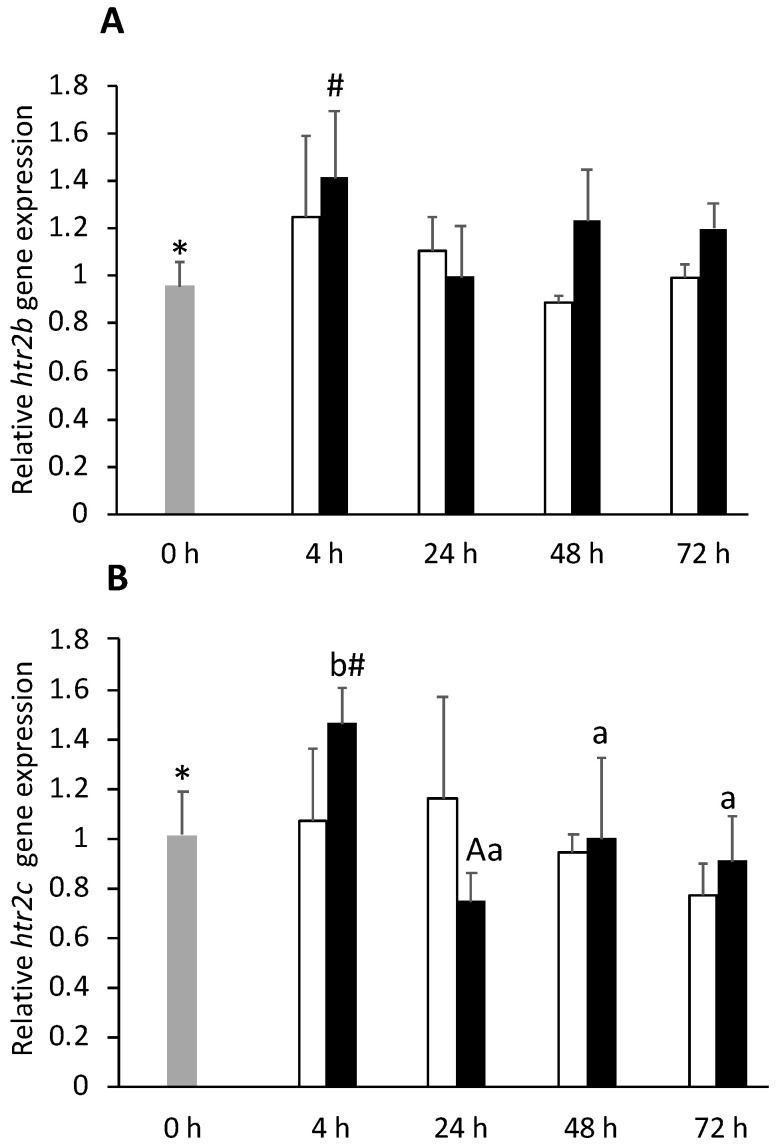
Telencephalic expression of serotonin receptors 2B (*htr2b*, **A**) and 2C (*htr2c*, **B**) in undisturbed European seabass (0 h, 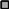) or i.p.-injected with a sham solution (CTRL, □) or Freund’s Incomplete Adjuvant (FIA, ∎) and sampled at 4, 24, 48, 72 h post-injection (means ± SD, n = 8). One-way ANOVA was performed to identify differences between i.p.-injected fish and the undisturbed group, followed by a Tukey post-hoc test. Different symbols (*, #) stand for significant differences between i.p.-injected groups and the undisturbed group (0 h). Two-way ANOVA was performed to identify significant differences within the i.p.-injected fish, followed by a Tukey post-hoc test. Capital letters stand for significant differences between stimuli. Lower-case letters indicate significant differences between sampling times (*p* ≤ 0.05).

Serotonin receptor 2A (*htr2b*) expression was upregulated in FIA-injected group, regardless of sampling point (Supplementary File, Table S1). Telencephalic expression of *htr2b* and serotonin receptor 2C (*htr2c*) was higher in FIA-injected fish at 4 h than in 0 h group (Figure 1A,B respectively). FIA-injected fish enhanced serotonin receptor 1Aβ (*htr1aβ*) expression levels with respect to CTRL at 48 h (Supplementary File, Table S1). Glucocorticoid receptor 2 (*gr2*) was downregulated at 72 h in both injected groups (Supplementary File, Table S1). No significant differences between FIA, CTRL, and 0 h groups were observed regarding glucocorticoid receptor 1 (*gr1*) and tryptophan hydroxylase 1 (*tph1*) gene expression, although both genes were downregulated over time, irrespective of treatment.

**Figure 2:**
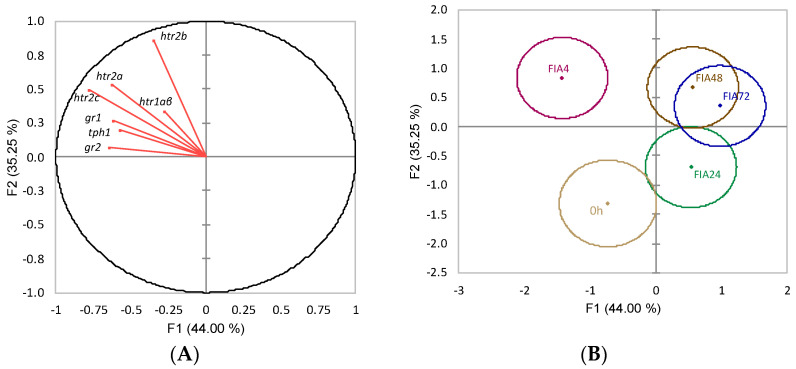
Canonical discriminant analysis of European seabass telencephalic expression of HPI-axis-related genes. (**A**) Correlation variables/factors (factor loads) for two main discriminant functions (F1 and F2); *gr1*, glucocorticoid receptor 1; *gr2*, glucocorticoid 2; *htr1aβ*, serotonin receptor 1Aβ; *htr2a*, serotonin receptor 2A; *htr2b*, serotonin receptor 2B; *htr2c*, serotonin receptor 2C; *tph1*, tryptophan hydroxylase 1. (**B**) Canonical discriminant scores of each group. Group centroids are marked by a small diamond, whereas circles indicate data distribution per group.

When evaluating linear functions of HPI-related variables in the telencephalon and their contributions to differences between 0 h and FIA groups (FIA4, FIA24, FIA48, FIA72), the overall discriminant analysis performance was very reasonable (Wilks λ = 0.19, *p* = 0.04). It resulted in four discriminant functions, with the first two accounting for 79.2% of the total variability (Figure 2A). The first discriminant function (F1, 44%) was negatively loaded by *gr1*, *gr2*, *htr2a,* and *htr2c* (i.e., lower gene expression) (Figure 2A, correlations of −0.62, −0.64, −0.62, −0.78, respectively) whereas the second function (F2, 35.25%) was positively loaded by *htr2b* (i.e., higher gene expression) (Figure 2A, correlation of 0.86). The analysis of Mahalanobis distances between groups’ multivariate means demonstrated that FIA4, FIA48, and FIA72 differed from 0 h, and that FIA4 differed from FIA24 and FIA72 (*p* < 0.05, Figure 2B).

#### 3.1.2. Optic Tectum

**Figure 3:**
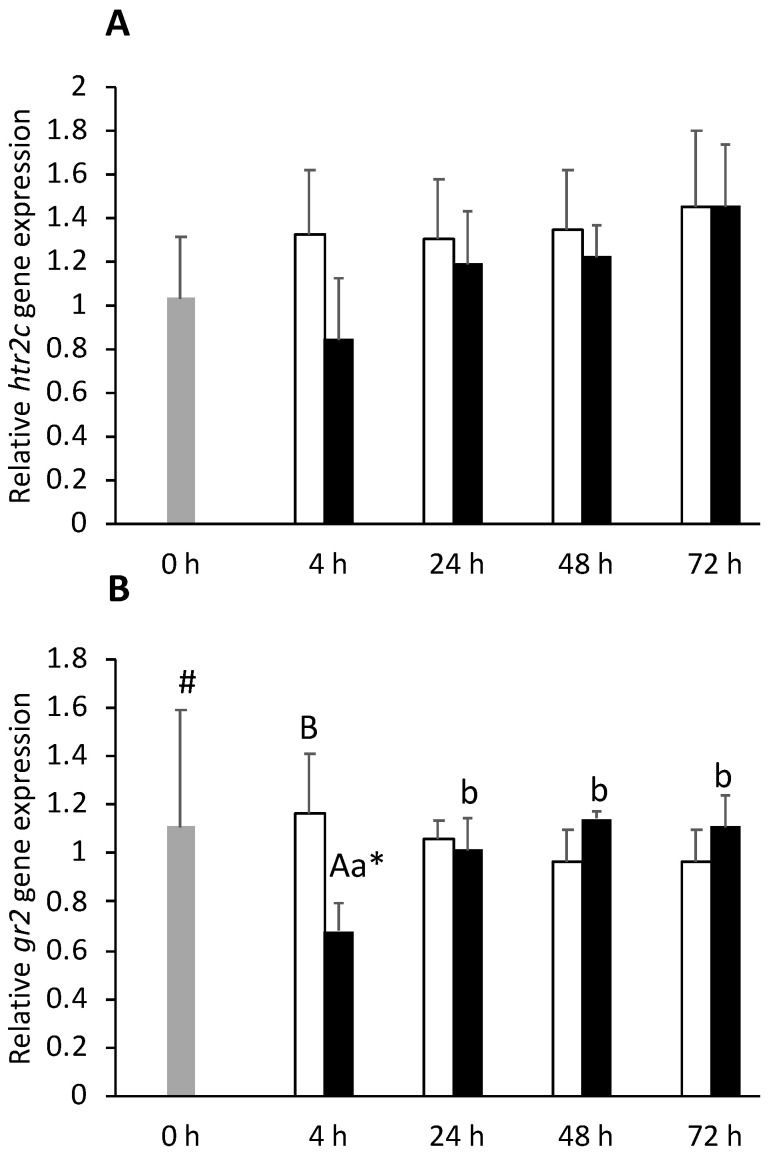
Optic tectum expression of serotonin receptor 2C (*htr2c*, **A**) and glucocorticoid receptor 2 (*gr2*, **B**) in undisturbed European seabass (0 h, 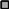) or i.p.-injected with a sham solution (CTRL, □) or Freund’s Incomplete Adjuvant (FIA, ∎) and sampled at 4, 24, 48, 72 h post-injection (means ± SD, n = 8). Different symbols (* and #) stand for significant differences between i.p.-injected groups and the undisturbed group (0 h). Capital letters stand for significant differences between stimuli. Lower-case letters indicate significant differences between sampling times. Further details in legend of Figure 1.

An extended gene expression suppression was observed in the FIA group compared to the CTRL group, regardless of the sampling point. Similar to *htr2c* (Figure 3A), *gr1*, *htr1aβ*, and *htr2a* were all downregulated (Supplementary File, Table S2). In the same way, *gr2* gene expression decreased in FIA-injected fish at 4 h compared to both 0 h and CTRL groups (Figure 3B). No significant differences were observed regarding both *htr2b* and *tph1*.

**Figure 4:**
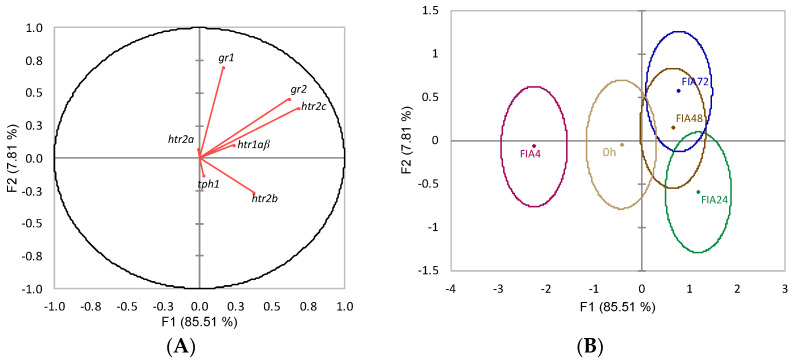
Canonical discriminant analysis of European seabass optic tectum expression of HPI-axis-related genes. (**A**) Correlation variables/factors (factor loads) for two main discriminant functions (F1 and F2); *gr1*, glucocorticoid receptor 1; *gr2*, glucocorticoid 2; *htr1aβ*, serotonin receptor 1Aβ; *htr2a*, serotonin receptor 2A; *htr2b*, serotonin receptor 2B; *htr2c*, serotonin receptor 2C; *tph1*, tryptophan hydroxylase 1. (**B**) Canonical discriminant scores of each group. Group centroids are marked by a small diamond, whereas circles indicate data distribution per group.

The discriminant analysis to neuroendocrine-related genes was also statistically significant (Wilks λ = 0.27, *p* = 0.04), with the first two discriminant functions explaining 93.3% of the data total variability (Figure 4). The first discriminant function (F1, 85.5%) was positively loaded by both *gr2* and *htr2c* (i.e., higher expression) (Figure 4A, correlations of 0.62 and 0.68, respectively), whereas the second function (F2, 7.8%) was positively loaded by *gr1* (i.e., higher expression) (Figure 4A, correlation of 0.70). The analysis of Mahalanobis distances between groups’ multivariate means demonstrated that FIA4 was significantly different from FIA24, FIA48, and FIA72 (*p* < 0.05, Figure 4B).

#### 3.1.3. Hypothalamus

**Figure 5:**
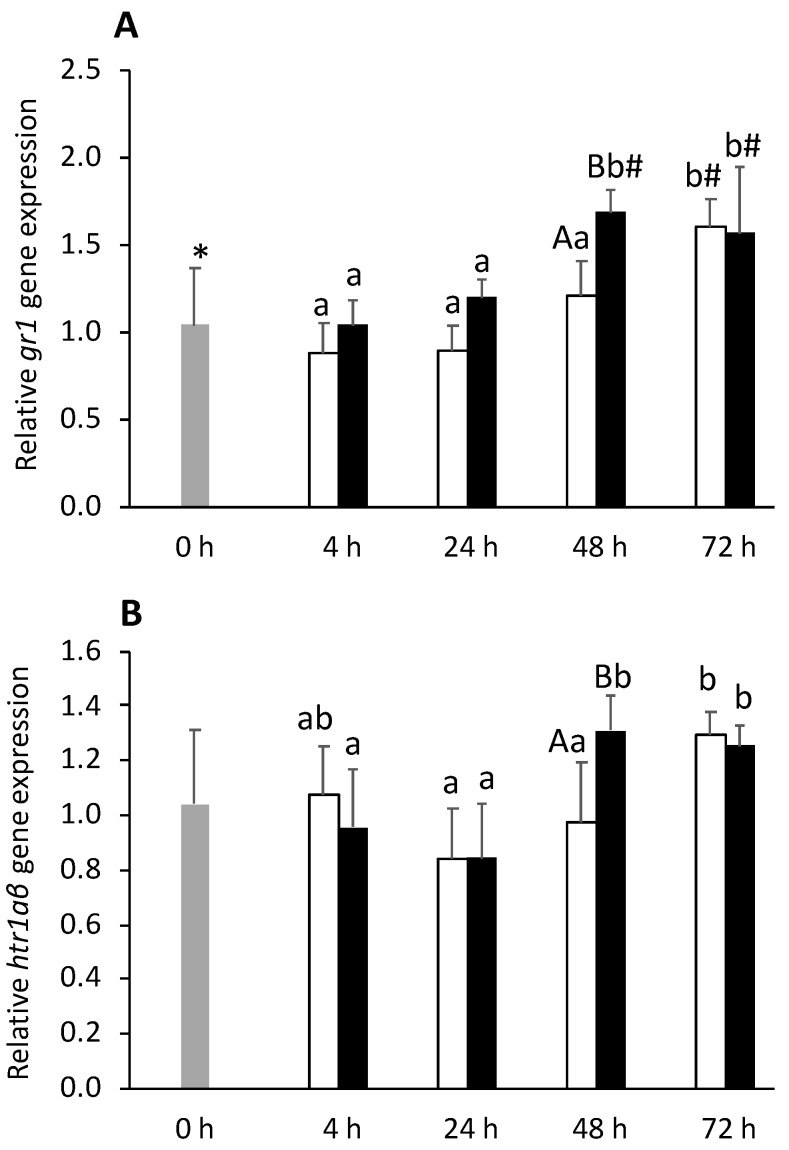
Hypothalamic expression of glucocorticoid receptor 1 (*gr1*, **A**) and serotonin receptor 1A*β* (*htr1aβ*, **B**) in undisturbed European seabass (0 h, 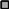) or i.p.-injected with a sham solution (CTRL, □) or Freund’s Incomplete Adjuvant (FIA, ∎) and sampled at 4, 24, 48, 72 h post-injection (means ± SD, n = 8). Different symbols (* and #) stand for significant differences between i.p.-injected groups and the undisturbed group (0 h). Capital letters stand for significant differences between stimuli. Lower-case letters indicate significant differences between sampling times. Further details in legend of Figure 1.

Gene expression of *gr1* was upregulated by i.p. injection, being higher at 48 h in the FIA group, compared to the CTRL group (Figure 5A). A similar feature was observed for *gr2* expression (data not shown). In addition, corticotropin-releasing hormone (*crh*) expression was upregulated by i.p. injection whereas *tph1* transcription significantly decreased (Supplementary File, Table S3). Expression levels of *htr1aβ* were higher at 48 h in the FIA group compared to the CTRL group (Figure 5B). No significant differences between the FIA and CTRL groups were detected regarding corticotropin-releasing hormone-binding protein (*crhbp*) and *tph1* gene expression (Supplementary File, Table S3).

**Figure 6:**
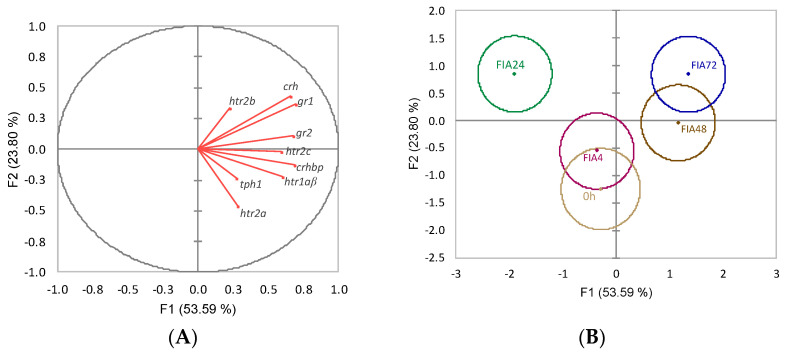
Canonical discriminant analysis of European seabass hypothalamic expression of HPI-axis-related genes. (**A**) Correlation variables/factors (factor loads) for two main discriminant functions (F1 and F2); *gr1*, glucocorticoid receptor 1; *gr2*, glucocorticoid 2; *htr1aβ*, serotonin receptor 1Aβ; *htr2a*, serotonin receptor 2A; *htr2b*, serotonin receptor 2B; *htr2c*, serotonin receptor 2C; *tph1*, tryptophan hydroxylase 1; *crh*, corticotropin-releasing hormone; *crhbp*, crh-binding protein. (**B**) Canonical discriminant scores of each group. Groups centroids are marked by a small diamond, whereas circles indicate data distribution per group.

Neuroendocrine variables discriminant analysis (Wilks λ = 0.12, *p* = 0.002) produced four discriminant functions from which the first two accounted for 77.4% of the total dataset variability (Figure 6). The first function (F1, 53.6%) was positively loaded by *gr1*, *gr2*, *crh*, *crhbp*, and *htr1aβ* (i.e., higher expression) (Figure 6A, correlations of 0.70, 0.68, 0.66, 0.69, and 0.60, respectively) while no significant loadings were attributed to the second function (F2, 23.8%).The analysis of Mahalanobis distances between groups’ multivariate means demonstrated that the t0h group was significantly different from FIA24 and FIA72, FIA4 was different from FIA72, and FIA24 differed from FIA48 and FIA72 (*p* < 0.05, Figure 6B).

#### 3.1.4. Pituitary Gland

**Figure 7:**
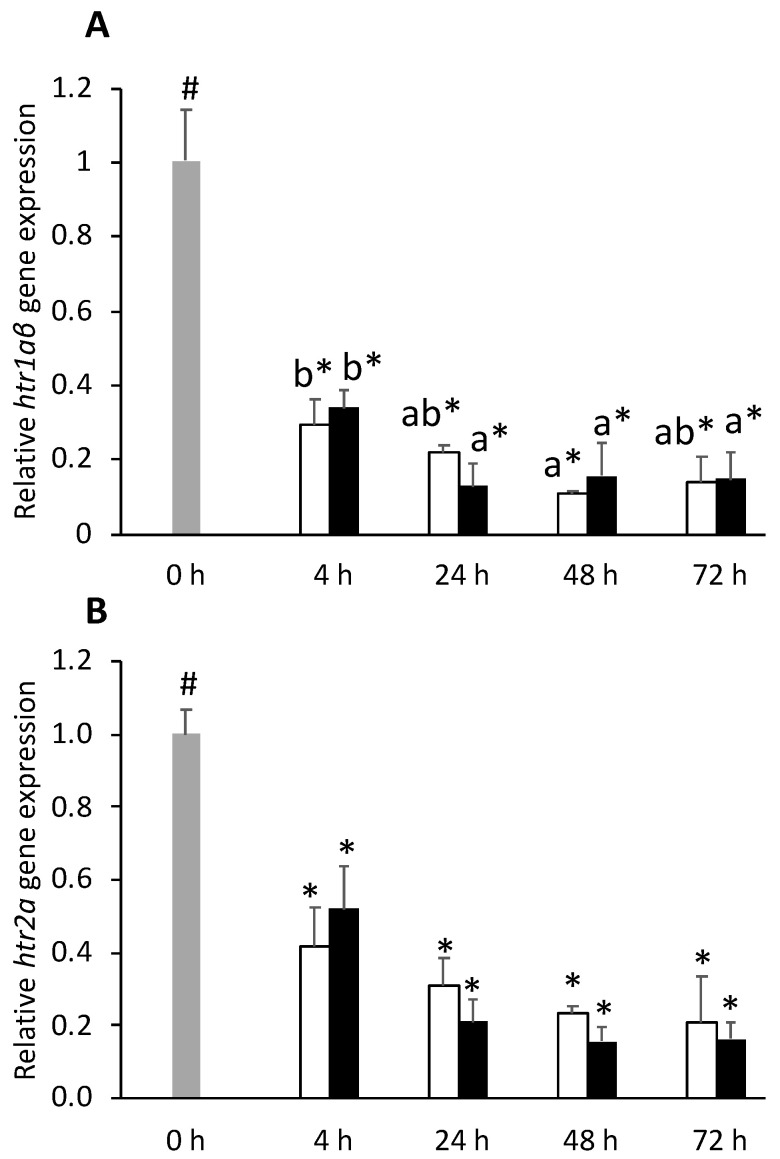
Pituitary gland expression of serotonin receptor 1Aβ (*htr1aβ*, **A**) and serotonin receptor 2A (*htr2a*, **B**) in undisturbed European seabass (0 h, 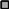) or i.p.-injected with a sham solution (CTRL, □) or Freund’s Incomplete Adjuvant (FIA, ∎) and sampled at 4, 24, 48, 72 h post-injection (means ± SD, n = 8). Different symbols (* and #) stand for significant differences between i.p.-injected groups and the undisturbed group (0 h). Capital letters stand for significant differences between stimuli. Lower-case letters indicate significant differences between sampling times. Further details in legend of Figure 1.

Serotonin receptors *htr1aβ* and *htr2a* were downregulated in the pituitary gland of all injected fish, at all time-points (Figure 7A,B, respectively). Expression level of *htr2c* was downregulated in FIA-injected fish sampled at 24 h compared to the 0 h group and was also lower in this group respect to CTRL regardless of sampling time (Supplementary File, Table S4).

**Figure 8:**
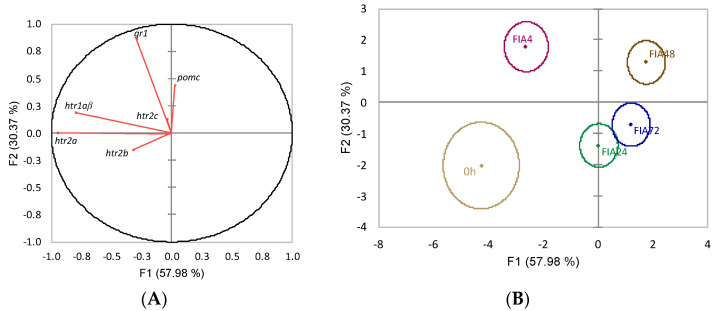
Canonical discriminant analysis of European seabass pituitary gland expression of HPI-axis-related genes. (**A**) Correlation variables/factors (factor loads) for two main discriminant functions (F1 and F2); *gr1*, glucocorticoid receptor 1; *htr1aβ*, serotonin receptor 1Aβ; *htr2a*, serotonin receptor 2A; *htr2b*, serotonin receptor 2B; *htr2c*, serotonin receptor 2C; *pomc*, proopiomelanocortin. (**B**) Canonical discriminant scores of each group. Group centroids are marked by a small diamond, whereas circles indicate data distribution per group.

The discriminant analysis to neuroendocrine variables had an overall satisfactory performance (Wilks λ = 0.43, *p* = 0.028), and the first two discriminant functions accounted for 88.4% of total data variability (Figure 8). The first function (F1, 58%) was negatively loaded by *htr1aβ* and *htr2a* (i.e., lower expression) (Figure 8A, correlations of −0.80 and −0.95, respectively) whereas the second discriminant function (F2, 30.4%) was positively loaded by *gr1* (i.e., higher expression) (Figure 8A, correlation of 0.86). The analysis of Mahalanobis distances showed that group 0 h was significantly different from all FIA groups, FIA4 was different from FIA24, FIA48 and FIA72, and FIA24 also different from FIA48 (*p* ≤ 0.05, Figure 8B).

### 3.2. Opioid Receptors Response

#### 3.2.1. Telencephalon

**Figure 9:**
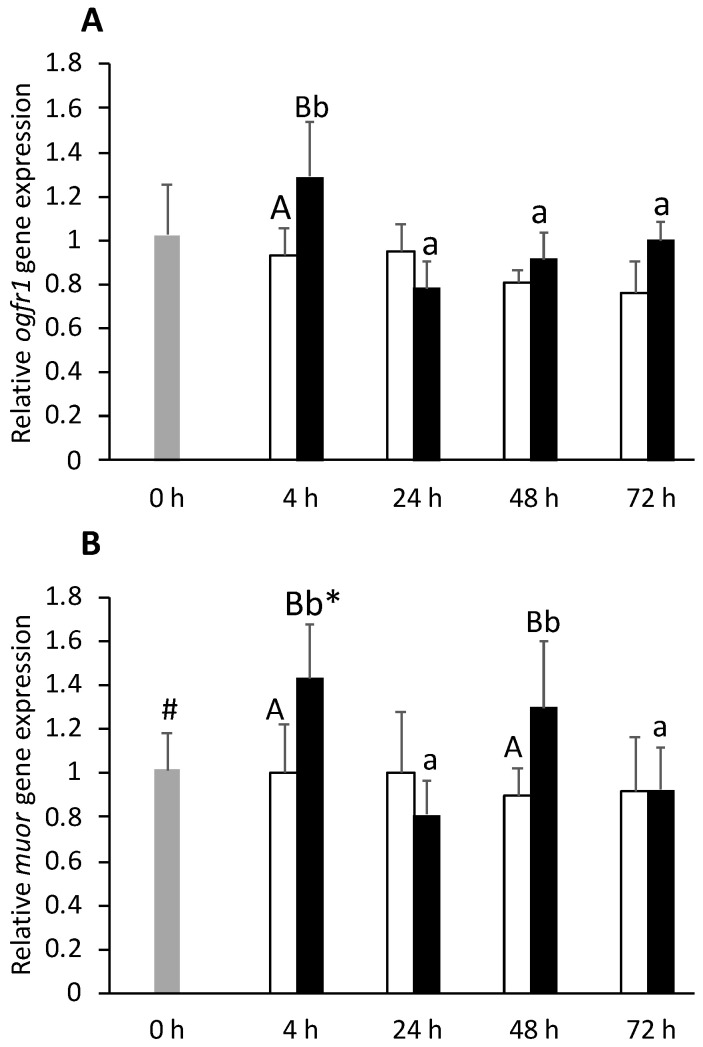
Telencephalic expression of opioid growth factor 1 (*ogfr1*, **A**) and mu opioid receptors (*muor*, **B**) in undisturbed European seabass (t0h, 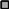) or i.p.-injected with a sham solution (CTRL, □) or Freund’s Incomplete Adjuvant (FIA, ∎) and sampled at 4, 24, 48, 72 h post-injection (means ± SD, n = 8). Different symbols (* and #) stand for significant differences between i.p.-injected groups and the undisturbed group (0 h). Capital letters stand for significant differences between stimuli. Lower-case letters indicate significant differences between sampling times. Further details in legend of Figure 1.

Intra-peritoneal injection suppressed nociception receptor (*nopr*) gene expression in the telencephalon of CTRL and FIA groups from 24 h post-injection until the end of the experiment (Supplementary File, Table S1). The inflammatory condition enhanced opioid growth factor receptor 1 (*ogfr1*) gene expression at 4 h respect to the CTRL group (Figure 9A), but expression levels significantly decreased at 4 h post-injection to values similar to those of the CTRL group. The mu opioid receptor (*muor*) expression was similarly upregulated in FIA-injected fish, in which expression was higher than that of the CTRL group at both 4 and 48 h post-injection (Figure 9B). At 4 h, *muor* expression was also higher in FIA than 0 h. Finally, the delta opioid receptor 2 (*dor2*) increased also significantly in fish under inflammation respect to CTRL fish, regardless of sampling time (Supplementary File, Table S1).

**Figure 10:**
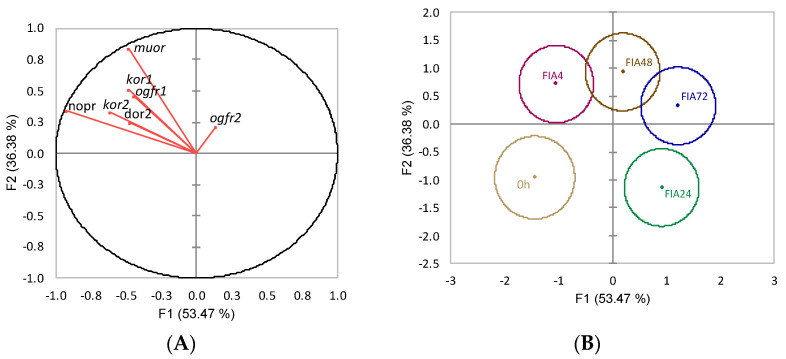
Canonical discriminant analysis of European seabass telencephalic expression of opioid receptor genes. (**A**) Correlation variables/factors (factor loads) for two main discriminant functions (F1 and F2); *muor*, mu opioid receptor; *kor1*, kappa opioid receptor 1; *kor2*, kappa opioid receptor 2; *dor2*, delta opioid receptor 2; *ogfr1*, opioid growth factor receptor 1, *ogfr2*, opioid growth factor receptor 2; *nopr*, nociceptin opioid receptor. (**B**) Canonical discriminant scores of each group. Group centroids are marked by a small diamond, whereas circles indicate data distribution per group.

The discriminant analysis for opioid receptors’ gene expression of 0 h and FIA groups (Wilks λ = 0.2, *p* = 0.004) resulted in four linear functions from which the first two accounted for 89.8% of the data total variability (Figure 10). The first discriminant function (F1, 53.5%) was negatively loaded by kappa opioid receptor 2 (*kor2*) and nociceptin opioid receptor (*nopr*) (i.e., lower gene expression) (Figure 10A, correlations of −0.61 and −0.93, respectively) while the second function (F2, 36.4%) was positively loaded by *muor* (i.e., higher gene expression) (Figure 10A, correlation of 0.85%). The analysis of Mahalanobis distances between group’s multivariate means showed that t0h was different from FIA24, FIA48, and FIA72. The FIA4 group was different from both FIA24 and FIA72. FIA24 was also different from FIA48 (*p* < 0.05, Figure 10B).

#### 3.2.2. Optic Tectum

**Figure 11:**
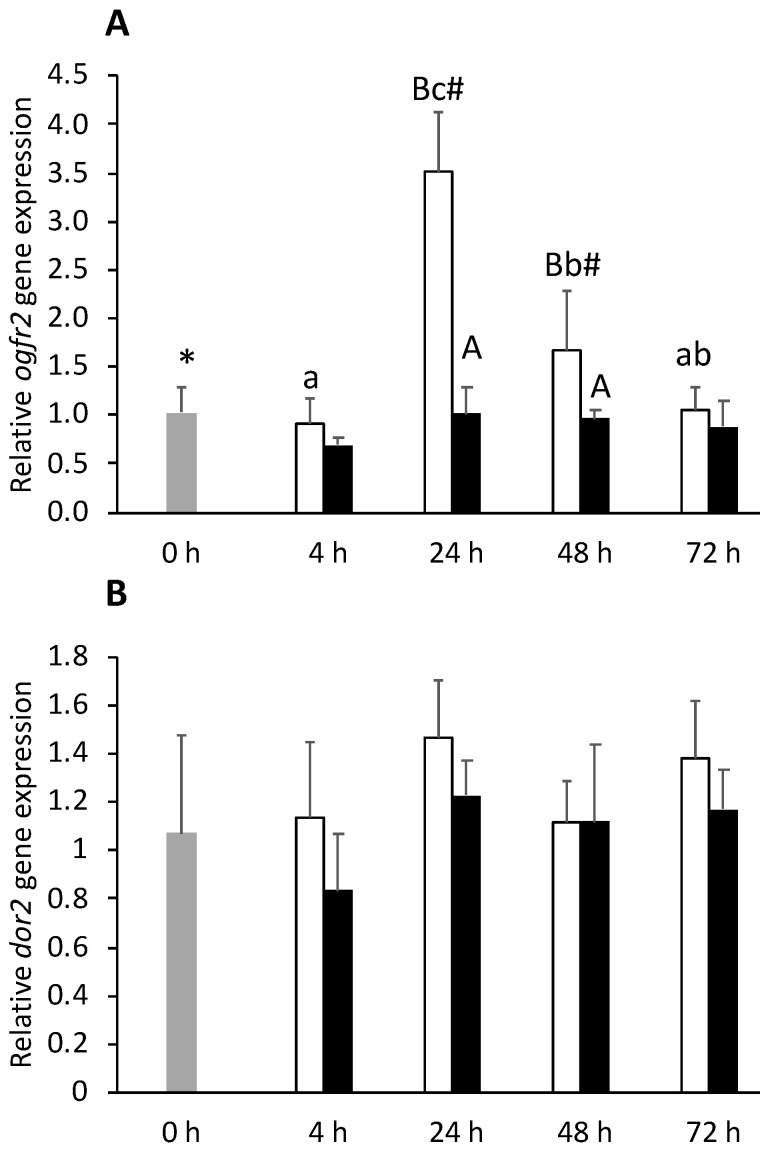
Optic tectum expression of opioid growth factor receptor 2 (*ogfr2*, **A**) and delta opioid receptor 2 (*dor2*, **B**) in undisturbed European seabass (0 h, 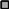) or i.p.-injected with a sham solution (CTRL, □) or Freund’s Incomplete Adjuvant (FIA, ∎) and sampled at 4, 24, 48, 72 h post-injection (means ± SD, n = 8). Different symbols (* and #) stand for significant differences between i.p.-injected groups and the undisturbed group (0 h). Capital letters stand for significant differences between stimuli. Lower-case letters indicate significant differences between sampling times. Further details in legend of Figure 1.

In the optic tectum, inflammation seemed to carry out a transversal inhibitory effect where *ogfr1* (at 4 h post-injection, Supplementary File, Table S2) and opioid growth factor receptor 2 (*ogfr2*) (at 24 and 48 h post-injection, Figure 11A) expression levels decreased in FIA-injected fish compared to CTRL fish. In addition, *kor2*, *muor,* and *dor2* were also downregulated in FIA fish, compared to CTRL fish, regardless of sampling point (Figure 11B). Moreover, *nopr* expression was lower in the FIA group than in CTRL group at both 4 and 24 h post-injection (Supplementary File, Table S2).

The performance of the discriminant analysis to opioid receptors gene expression showed no significant differences amongst data variability (Wilks λ = 0.4, *p* = 0.13).

#### 3.2.3. Hypothalamus

**Figure 12:**
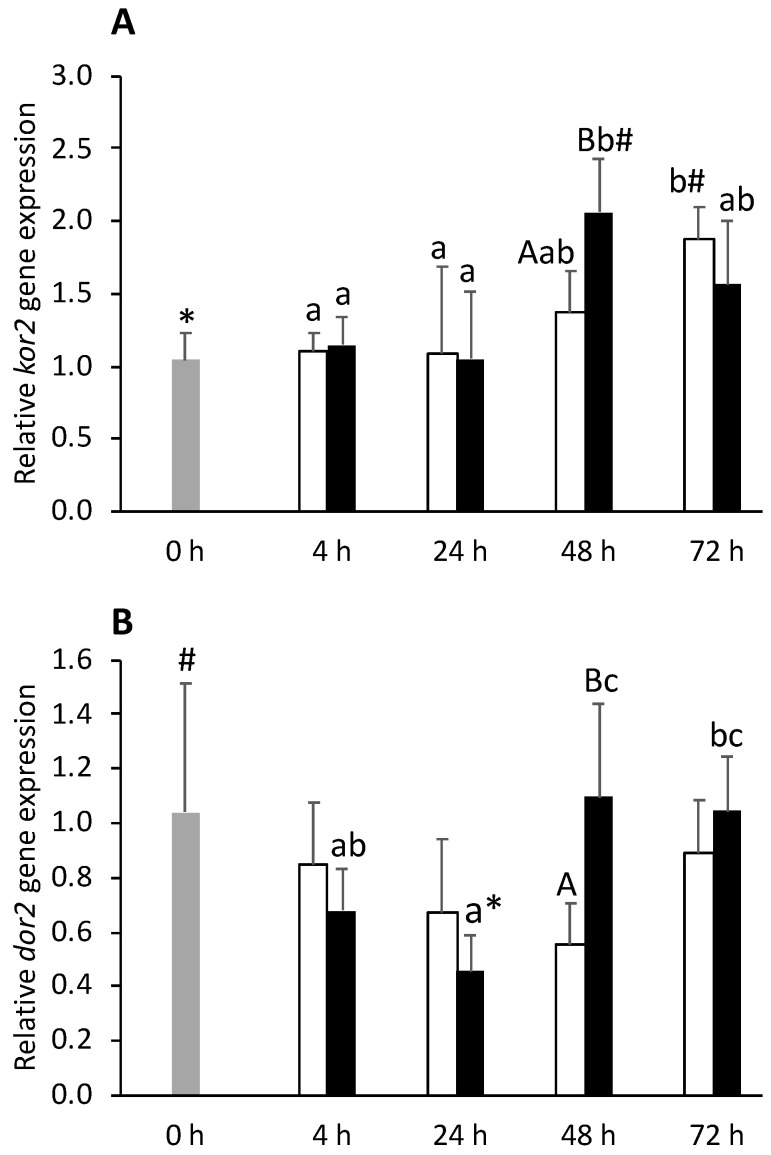
Hypothalamic expression of kappa opioid receptor 2 and delta opioid receptor 2 (*kor2*, **A** and *dor2*, **B**) in undisturbed European seabass (0 h, 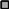) or i.p.-injected with a sham solution (CTRL, □) or Freund’s Incomplete Adjuvant (FIA, ∎) and sampled at 4, 24, 48, 72 h post-injection (means ± SD, n = 8). Different symbols (* and #) stand for significant differences between i.p.-injected groups and the undisturbed group (0 h). Capital letters stand for significant differences between stimuli. Lower-case letters indicate significant differences between sampling times. Further details in legend of Figure 1.

Expression levels of both *ogfr1* and *kor2* were upregulated by inflammation whereas *muor* was downregulated. For the three affected transcripts, hypothalamic reaction to i.p. injection was earlier in the FIA group compared to CTRL group responses. Fish undergoing an inflammatory response increased expression levels of *ogfr1*, *kor2* (Figure 12A), and *dor2* (Figure 12B) in FIA fish at 48 h compared to their CTRL counterparts. Differently, it downregulated *dor2* transcription at 24 h respect to 0 h fish (Figure 12B).

**Figure 13:**
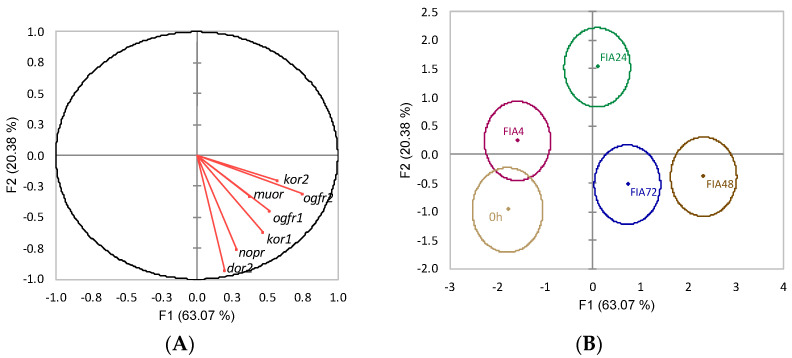
Canonical discriminant analysis of European seabass hypothalamic expression of opioid receptor genes. (**A**) Correlation variables/factors (factor loads) for two main discriminant functions (F1 and F2); *muor*, mu opioid receptor; *kor1*, kappa opioid receptor 1; *kor2*, kappa opioid receptor 2; *dor2*, delta opioid receptor 2; *ogfr1*, opioid growth factor receptor 1, *ogfr2*, opioid growth factor receptor 2; *nopr*, nociceptin opioid receptor. (**B**) Canonical discriminant scores of each group. Group centroids are marked by a small diamond, whereas circles indicate data distribution per group.

The discriminant analysis (Wilks λ = 0.09, *p* < 0.0001) produced four discriminant functions from which the first two accounted for 83.5% of the total dataset variability (Figure 13). The first function (F1, 63.1%) was positively loaded by *ogfr2* (i.e., higher expression) (Figure 13A, correlation of 0.75) while the second function (F2, 20.4%) was negatively loaded by kappa opioid receptor 1 (*kor1*), *nopr*, and *dor2* (i.e., lower gene expression) (Figure 13A, correlations of −0.62, −0.76, and −0.93, respectively). The Mahalanobis distances between groups’ multivariate means showed that t0h and FIA4 groups were significantly different from FIA24, FIA48, and FIA72, but not different from one another. Furthermore, FIA24 differed from FIA48 and FIA72, while FIA48 was also different from FIA72 (*p* < 0.05, Figure 13B).

#### 3.2.4. Pituitary Gland

**Figure 14:**
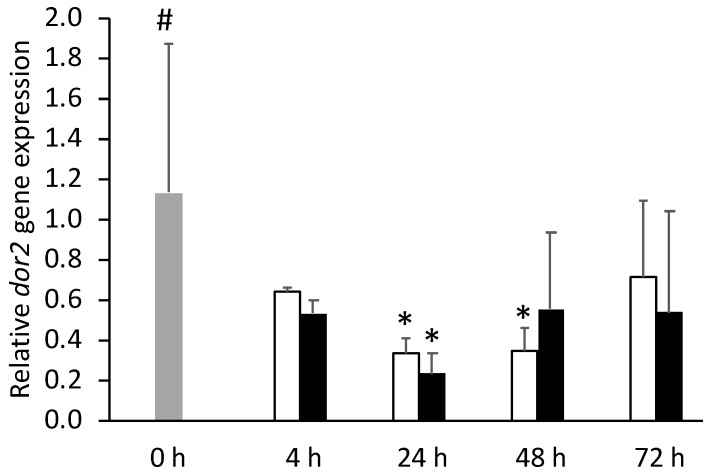
Pituitary gland expression of delta opioid 1 (*dor2*) in undisturbed European seabass (0 h, 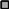) or i.p.-injected with a sham solution (CTRL, □) or Freund’s Incomplete Adjuvant (FIA, ∎) and sampled at 4, 24, 48, 72 h post-injection (means ± SD, n = 8). Different symbols (* and #) stand for significant differences between i.p.-injected groups and the undisturbed group (0 h). Further details in legend of Figure 1.

In the pituitary gland, *dor2* gene expression was downregulated at 24 h in both injected groups (Figure 14). Otherwise, no effects of inflammation were detected in this tissue opioid receptors gene expression.

**Figure 15:**
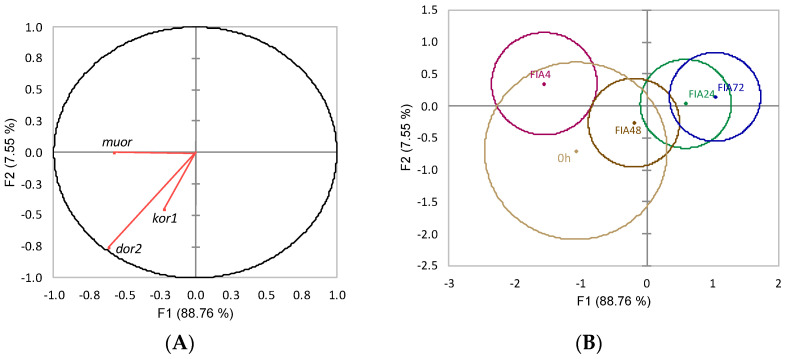
Canonical discriminant analysis of European seabass pituitary gland expression of opioid receptor genes. (**A**) Correlation variables/factors (factor loads) for two main discriminant functions (F1 and F2); *muor*, mu opioid receptor; *kor1*, kappa opioid receptor 1; *dor2*, delta opioid receptor 2. (**B**) Canonical discriminant scores of each group. Groups centroid are marked by a small diamond, whereas circles indicate data distribution per group.

The first two linear functions resulting from data discriminant analysis (Wilks λ = 0.04, *p* < 0.03) explained 96.3% of the data variability (Figure 15). Both functions were negatively loaded by *dor2* (i.e., lower gene expression) (Figure 15A, correlations values of −0.61 and −0.75, respectively). The analysis of Mahalanobis distances between groups’ multivariate means showed that the FIA4 group was significantly different from and FIA24 and from FIA72 (*p* ≤ 0.05, Figure 15B).

## 4. Discussion

Fish handling is amongst the top stress-inducing procedures in aquaculture. Chasing and intra-peritoneally injecting fish, which involves air exposure, is a procedure often carried out during vaccination. It represents an acute stress that activates the HPI-axis and initiates a neuroendocrine response, irrespective of potential long-term physiological consequences [22,23]. In this study, while being aware of the acute stress (i.p. injection and/or air exposure) implications on brain gene expression, there was no intention to evaluate such effects. However, innate immune mechanisms are also triggered right after the immune challenge (FIA i.p. injection). Hence, our choice of sampling at such an early time point (4 h) implies that it is likely to witness some lingering stress effects (induced artifact due to injection procedure). Thus, they might mask those inflicted by the development of the inflammatory response at this early stage.

### 4.1. HPI-Axis Response

A stress-induced effect was observed in both experimental groups when *crh*, encoding for CRH—one of the first compounds to be released by the hypothalamic tissue upon neuroendocrine stimulation [7]—was found upregulated at 72 h post-injection. However, our sampling scheme most certainly missed a much earlier induction, previously demonstrated by Skrzynska and co-workers [22]. One would then expect rising plasma ACTH levels due to CRH-induced pituitary secretion of this hormone [7]. Despite the fact that we did not measure it directly, expression of *pomc* (ACTH genetic precursor) was evaluated in the pituitary gland but acute stress did not change its transcriptional rate. Transcription of *pomc* is regulated by several factors, including CRH (activation) and GR (impairment) [24]. GR1 was down-regulated in the pituitary gland at 72 h, which would increase the expectations of observing *pomc* upregulation. Nonetheless, the absence of *pomc* transcriptional changes does not imply that there was no induction of ACTH response, since an expression enhancement could have occurred at an earlier time. Indeed, Liu and co-workers have observed the upregulation of *pomcb* in the pituitary gland of gilthead seabream, one hour following air exposure, with gene expression returning to basal levels at 6 and 24 h post stress [23]. Yet, transcription of *pomca* was not significantly altered by the acute stress, pointing at potentially different isoform functions. In the present study, the coding sequence selected for this gene does not specify to a particular isoform, but it shows high similarity degree to other teleost species’ *pomca* nucleotide sequences.

There are contrasting reports of stress and cortisol modulatory effects in which GR transcriptomic regulation is concerned [25,26,27,28]. In the head-kidney of European seabass leucocytes, both *gr1* and *gr2* were upregulated upon in vitro cortisol treatment [28]. The present study shows that these genes’ behavior towards acute stress was the opposite. Specifically, *gr1* was upregulated in the hypothalamus, whereas the telencephalic *gr2* transcription was downregulated. There is a great gap in the knowledge of brain glucocorticoid receptors’ distribution and dynamics in teleost fish, with most studies focusing on other tissues [28,29,30]. This central localization, where neuroendocrine pathways are initiated, indicates their involvement in mediating corticosteroid feedback mechanisms through transcriptomic regulation. The hypothalamic stress-responsive *gr1* enhancement expression appears to be a regulatory mechanism triggered upon neuroendocrine stimulation. Accordingly, it was concomitant to *crh* higher expression levels in the same tissue.

In parallel to the stimulation at the central nervous system of the neuroendocrine response, acute stress also leads to a rise in monoaminergic activity. Nevertheless, these changes are known to occur almost immediately after the acute stress (chasing/handling/injection), with levels typically returning to basal values within 4–8 h post-stress [31]. At 4 h post-injection, two of the evaluated serotonin receptors (*htr1aβ* and *htr2a*) were markedly downregulated in the pituitary gland. Moreover, in the hypothalamus, i.p. injection inhibited the gene expression of the serotonin synthesizing enzyme, *tph1*. Despite this being in opposition to what was observed by Gesto and co-workers [31], a similar inhibition of *htr1aα* expression was observed in the telencephalon of rainbow trout (*Oncorhynchus mykiss*) 4 h after being subjected to acute stress (confinement, [32]). Gene expression data from this study do not allow to discriminate auto- from heteroreceptors. Nevertheless, and regardless of the extension and direction of this modulatory effect (which was not within the scope of this study), stress-induced downregulation of these receptors’ gene expression, together with a decreased availability of serotonin synthesizing enzyme, suggests that the neuroendocrine response at some level regulates serotonergic activity. In support of this hypothesis, Medeiros and McDonald [33] showed in Gulf toad-fish that *htr1a* was downregulated by cortisol treatment, indicating that serotonin receptors were under negative feedback control of this hormone.

Intraperitoneal injection with a phlogistic agent elicits a local inflammatory response (FIA-injected fish). Overall some of these genes’ behaviour was inverted while others were unaltered. What was remarkably clear was a regionalization of the brain response, i.e., establishing marked response patterns (stimulation vs. inhibition) according to different isolated regions. In what the telencephalon is concerned, i.p., injection with FIA induced the expression of three serotonin receptors, two of them (*htr2b* and *htr2c*) at 4 h post-injection. Note that in mammals, this part of the brain includes the so-called limbic system consisting of structures that support several functions such as emotion, behaviour, and long-term memory. It is vastly innervated by serotonergic neurons coming from the raphe nuclei, and it interacts with the mammalian HPI-axis homolog, the hypothalamus-pituitary-adrenal axis [34]. In teleosts, an evident connection between telencephalic serotonin and HPI-axis has been demonstrated too [34,35,36,37]. Yet, little is known about the effect of peripheral immune signals on serotonergic pathways. The fact that there was an early induction of these serotonin receptors following the inflammatory insult might be related to circulating immune mediators such as cytokines, which are intensively produced at the onset of an immune response and have also been associated to the activation of mammalian central neuroendocrine pathways, including serotonergic pathways [10,38].

In line with the stimulatory pattern observed in the telencephalic region, the development of inflammatory response was accompanied by upregulation of both glucocorticoid receptors (*gr1* and *gr2*) and *htr1aα* genes in the hypothalamus at 48 h post-injection. This was a response unexpectedly delayed in time considering these are mechanisms generally known to be mounted soon after the initial trigger [39], even when the trigger is an immune mediator (which begins to be synthesized at the onset of the inflammatory response [40]). Interestingly, this seemed to be a critical time point for the hypothalamic response, given several genes were differently expressed between control and FIA-injected fish at this time (including opioid-related genes). The ANOVA statistical approach did not retrieve significant alterations on the more obvious *crh* or *crhbp* genes. However, the discriminant analysis of data from the hypothalamus of fish undergoing inflammation attributed a significant role to these two genes which, together with both glucocorticoids (*gr1* and *gr2*) and one serotonin receptor (*htr1aβ*), showed time-dependent increased expression. Altogether, it is important to be aware of the effects of an acute stress, such as a peritoneal injection and/or air exposure, particularly at this first stage of the HPI-axis physiology. The absence of a more rapid and stronger hypothalamic response might be explained by a masking effect of the intraperitoneal puncture.

In contrast to the observed in telencephalon and hypothalamus, the optic tectum reacted to immune signaling with a general inhibitory behaviour. Although this region is the primary visual center in the brain of the teleost [41], several studies (including non-mammalian) have demonstrated its involvement in other physiological mechanisms, such as the stress response [25,36,42]. In our experiment, both glucocorticoid receptors (*gr1* and *gr2*) and three serotonergic receptors (*htr1aβ*, *htr2a*, and *htr2c*) were less expressed in the optic tectum of fish undergoing an inflammatory response than control fish (injected with HBSS). Lower expression levels of these receptors suggest a general shutdown of glucocorticoid-mediated regulatory pathways as well as an impaired serotonergic activity. However, whether these are direct consequences of inflammatory signals and to what extent these changes affect other tectal functions is far from being understood. On matters of the teleostean brain function, information is scarce at best. However, these divergent responses amongst different brain regions indicate different functions of these tissues in order to face the same stimulus.

### 4.2. Opioid Receptors

Opioid neuropeptides are mainly synthesized at the central nervous system, but they are ubiquitously produced in the organism. Met-enkephalin, β-endorphin and dynorphin are produced from different gene precursors and bind to their specific receptors, except for met-enkephalin that may bind to delta opioid receptor, mu opioid receptor and the opioid growth factor receptor [43]. Similarly, opioid receptors are expressed in the teleost brain and other organs such as the head-kidney [17]. Recent findings further support their involvement in the immune response, demonstrating a direct effect of opioids on carp phagocyte immune function and immune-related gene expression [18,44]. On the other hand, opioid receptors expression has also been shown to be modulated by in vitro immune stimulation [17]. In mammals, opioids are mostly known to mediate nociception and mood and have long been studied regarding their clinical use as pain killers and their addiction properties [45]. However, there is quite a significant gap in fish regarding their central nervous system function since nociception is still a critically debated issue amongst fish biologists [15,46]. Even less effort has been put into understanding their main dynamics in response to peripheral immune stimuli.

As reviewed by Wei and Loh [47], *muor* transcriptional regulation was found to be mediated—among others—by endocrine factors, such as cytokines and interferon-γ, which demonstrates how sensitive these receptors are to immune stimulation in mammals. However, such knowledge has resulted from in vitro studies with immune-related cell lines, indicating that it is important to consider these receptors’ localization when evaluating their transcriptional dynamics. In the brain, opioid receptors transcription is therefore likely to be differently regulated, even within different regions. In the present study, FIA-induced inflammation evoked a more noticeable hypothalamic *ogfr1*, *kor2,* and *dor2* gene expression enhancement than induced by the i.p. injection itself but only 48 h after the injection onset of inflammation. In addition, in the telencephalon, *muor* and *ogfr1* expressions were upregulated in FIA-injected fish, but this altered pattern was observed earlier at 4 h post-injection. Therefore, the two regions’ reactivity to an immune challenge is differently characterized in receptor types and in the time it takes for their transcription rate to change, suggesting a differential role of telencephalon and hypothalamus after an inflammatory change. interestingly telencephalic serotonin receptors were, as aforementioned, similarly upregulated in parallel to the unfolding inflammatory process, suggesting that both receptor families (i.e., serotonin and opioids receptors) might be involved in the same neurologic response to immune signals. Indeed, Tao and Auberbach [48] have shown that opioid infusion into the rat brain induced a rise in extracellular serotonin, an effect blocked by a selective μ-receptor antagonist, demonstrating the existence of a close relationship between both systems, at least in mammals. However, further studies are necessary in order to demonstrate a similar relationship in teleost.

Tectum opioid receptors behaved differently from the telencephalon and the hypothalamus and certainly to the pituitary gland, seemingly unaffected by inflammation. Indeed, and concerning what was observed with other neuroendocrine-related genes, most of the responding opioid receptor genes in the optic tectum were downregulated by inflammatory signaling, including those upregulated elsewhere. Far more than just the main visual center, the optic tectum is believed to gather other sensory modalities (electroreception, infra-red sensitivity, mechanoreception, etc.), then convey the acquired and processed information to motor neurons. Thereby, it is also involved in reactive behavior [49]. The responsiveness of opioid receptors in this particular region suggests that opioid peptides might also modulate sensory function. Additionally, in an inflammatory setting, these potential modulatory pathways seem to be altered, and so might be the resulting sensory and behavior functions.

## 5. Conclusions

Understanding how the fish brain processes peripheral inflammatory signals and whether such signaling molecules might trigger possible reactions is of great importance in fish health and welfare. The current approach yielded new data on the effect of a peritoneal inflammation on brain neuroendocrine response. Changes in various gene transcriptional rates in different brain regions—and in spite of the i.p. injection-induced stress—demonstrate the role of immune signals as transcriptional factors. It also shows their potential to regulate several brain-originated physiological responses such as the HPI-axis, behavior, nociception, and sensory functions. Finally, opioid receptors and other genes more directly involved with the neuroendocrine response are differently expressed in the evaluated brain regions, possibly pointing out the existence of site-specific functions.

## Figures and Tables

**Table 1:** Forward and reverse primers for real-time PCR.

Acronym	GenBank	Eff ^2^	AT ^3^	Product Length ^4^	Forward Primer Sequence	Reverse Primer Sequence
*40s*	HE978789.1	108.8	60	79	TGATTGTGACAGACCCTCGTG	CACAGAGCAATGGTGGGGAT
*ef1α*	AJ866727.1	92.8	57	144	AACTTCAACGCCCAGGTCAT	CTTCTTGCCAGAACGACGGT
*gr1*	AY619996.1	114.19	60	100	AAATCTGCCTGGTGTGTTCC	TGCCCTTTCACTGCTCTCTT
*gr2*	AY549305.1	109.4	55	142	CTTCTACAGCACCAGCACCA	TCTCCTGTTTGACCACACCA
*crh*	JF274994.1	110.21	60	200	AACCCAAAACTCCCAGCAG	TGTTCCCAACTTTCCCTTGT
*crhbp*	MG832822.1	105.47	60	199	TGTCATCTCCCAGTCACCAG	GCCATTTCCTCCAAGCAAC
*pomc*	AY691808.1	101.98	60	158	TCTTCCTCCTCCTCTCCACA	CGCCTTCTCATCTCTTCAGG
*htr1aβ*	DLAgn_00119560 ^1^	102.0	60	176	GGAGCGTAAAACGGTGAAAA	TGGGGTTGAGGAGAGAGTTG
*htr2a*	DLAgn_00222310 ^1^	103.8	60	18	CCTCTGACCTCTGTCCCATC	ACTGAAATCGTCCACACTGC
*htr2b*	DLAgn_00148380 ^1^	109.4	60	165	ATTGCCCTCGTCACTGTTCT	GCTGTGTTGGATTGGCTTCT
*htr2c*	DLAgn_00037670 ^1^	118.0	60	195	CATCCGCAACCCCATAGAG	ACGAAGGAGCCAATCAGCAT
*tph1*	DLAgn_00154580 ^1^	107.0	62	114	CGCATAGACTTCACAACAGAGG	CAGCAGAGGGAGGTTCTTCA
*ogfr1*	DLAgn_00128530 ^1^	96.8	60	185	GTTGGGAATGGAGATGGAAA	GCTTCAGATTTTGGCTCAGG
*ogfr2*	DLAgn_00089660 ^1^	96.6	60	146	CTTGCCTTCCTGTCTCCAGT	CTTGTCTCGGTTTCCTTTGG
*kor1*	DLAgn_00007470 ^1^	89.7	60	249	TCTGGTGCTTGTGGTAGTCG	TGGCAGTCTCTGTGTCCTTG
*kor2*	DLAgn_00077520 ^1^	82.0	60	163	CTCGTCAGTGTCCCCGAAAC	CCCCCTTCAGTTTGGCCGAGAG
*nopr*	DLAgn_00125610 ^1^	97.5	60	106	CTCCTTTCTCATCCCTGTGG	GTTGCGGTCCTTTTCCTTG
*muor*	DLAgn_00015310 ^1^	99.8	60	240	GTCACCAGCACCCTACCATT	CGAGGAGAGAATCCAGTTGC
*dor2*	DLAgn_00062690 ^1^	108.1	60	81	CGCTTCTCGGTCTCCATAACT	GGTCTCATTACTACTTGAAG

^1^ Sequences obtained from databases dicLab v1.0c seabass genome. ^2^ Efficiency of PCR reactions were calculated from serial dilutions of tissue RT reactions in the validation procedure. ^3^ Annealing temperature (°C). ^4^ Amplicon (nt).

## Data Availability

All data is provided in the main text or Supplementary File.

## Supplementary Materials

The following supporting information can be downloaded at: https://www.mdpi.com/article/10.3390/biology11030364/s1, Table S1. Telencephalic expression of neuroendocrine genes in the European seabass i.p. injected with Freud’s Incomplete Adjuvant (FIA) or Hank’s Balanced Salt Solution (CTRL) and sampled at 4, 24, 48 or 72 h post injection; Table S2. Optic tectum expression of neuroendocrine genes in the European seabass i.p. injected with Freud’s Incomplete Adjuvant (FIA) or Hank’s Balanced Salt Solution (CTRL) and sampled at 4, 24, 48 or 72 h post injection; Table S3. Hypothalamic expression of neuroendocrine genes in the European seabass i.p. injected with Freud’s Incomplete Adjuvant (FIA) or Hank’s Balanced Salt Solution (CTRL) and sampled at 4, 24, 48 or 72 h post injection; Table S4. Pituitary gland expression of neuroendocrine genes in the European seabass i.p. injected with Freud’s Incomplete Adjuvant (FIA) or Hank’s Balanced Salt Solution (CTRL) and sampled at 4, 24, 48 or 72 h post injection.
