# Supplementary material for: Acute Inflammation Induces Neuroendocrine and Opioid Receptor Genes Responses in the Seabass Dicentrarchus labrax Brain
Source: Biology (Basel). 2022 Feb 24;11(3):364. doi: 10.3390/biology11030364 (PMC8945561; doi:10.3390/biology11030364)
Supplement: Supplementary file 1 [file biology-11-00364-s001.zip › biology-1583839-supplementary.pdf]

Supplementary Table S1. Telencephalic expression of neuroendocrine genes in the European seabass i.p. injected with Freud's Incomplete Adjuvant (FIA) or Hank's Balanced Salt Solution (CTRL) and sampled at 4, 24, 48 or 72 h post injection.

Parameters	0h		4h	24h	48h	72h
gr1	0.96 ± 0.09	CTRL	0.95 ± 0.11	0.86 ± 0.20	0.73 ± 0.10	0.74 ± 0.11
		FIA	1.12 ± 0.24	0.87 ± 0.24	0.89 ± 0.22	0.78 ± 0.11
gr2	1.06 ± 0.07#	CTRL	0.99 ± 0.17	0.99 ± 0.19	0.86 ± 0.10	0.77 ± 0.05 *
		FIA	1.06 ± 0.05	0.93 ± 0.13	0.91 ± 0.13	0.78 ± 0.04 *
htr1aβ	1.05 ± 0.37	CTRL	0.85 ± 0.19	0.79 ± 0.13	0.65 ± 0.13 A	0.97 ± 0.19
		FIA	1.04 ± 0.40 ab	0.67 ± 0.10 a	1.09 ± 0.16 Bb	0.96 ± 0.26 ab
htr2a	1.02 ± 0.22	CTRL	1.09 ± 0.27	1.06 ± 0.30	0.98 ± 0.13	0.76 ± 0.23
		FIA	1.24 ± 0.24	0.78 ± 0.13	1.15 ± 0.43	0.90 ± 0.15
htr2b	0.96 ± 0.13 *	CTRL	1.25 ± 0.34	1.10 ± 0.14	0.89 ± 0.02	0.99 ± 0.06
		FIA	1.41 ± 0.28 #	1.00 ± 0.21	1.23 ± 0.21	1.20 ± 0.11
htr2c	1.01 ± 0.18 *	CTRL	1.07 ± 0.29	1.17 ± 0.41 B	0.95 ± 0.07	0.77 ± 0.13
		FIA	1.46 ± 0.14 #b	0.75 ± 0.12 Aa	1.00 ± 0.33 a	0.91 ± 0.18 a
ogfr1	1.02 ± 0.23	CTRL	0.93 ± 0.12 A	0.95 ± 0.12	0.81 ± 0.05	0.76 ± 0.15
		FIA	1.29 ± 0.25 Bb	0.78 ± 0.12 a	0.91 ± 0.12 a	1.00 ± 0.09 a
ogfr2	1.07 ± 0.44	CTRL	0.84 ± 0.16 a	1.79 ± 1.00 Bb	1.06 ± 0.78 ab	1.34 ± 0.28 ab
		FIA	0.82 ± 0.24	0.81 ± 0.18 A	1.25 ± 0.32	1.14 ± 0.14
kor1	1.01 ± 0.15	CTRL	0.88 ± 0.10	0.96 ± 0.23	0.70 ± 0.14	0.88 ± 0.25
		FIA	1.07 ± 0.10 b	0.74 ± 0.10 a	0.94 ± 0.21 ab	0.87 ± 0.12 ab
kor2	1.04 ± 0.15	CTRL	0.82 ± 0.21	0.85 ± 0.26	0.70 ± 0.03	0.73 ± 0.31
		FIA	1.05 ± 0.19	0.75 ± 0.21	0.84 ± 0.09	0.69 ± 0.27
nopr	1.06 ± 0.06 #	CTRL	1.01 ± 0.26	0.68 ± 0.18 *	0.66 ± 0.10 *	0.59 ± 0.07 *
		FIA	0.95 ± 0.20	0.51 ± 0.02 *	0.78 ± 0.23	0.57 ± 0.13 *
muor	1.01 ± 0.17 *	CTRL	1.01 ± 0.21 A	1.00 ± 0.28	0.90 ± 0.13 A	0.92 ± 0.24
		FIA	1.43 ± 0.25 #Bb	0.81 ± 0.16 a	1.30 ± 0.30 Bb	0.92 ± 0.19 a
dor2	0.95 ± 0.23	CTRL	0.69 ± 0.31	0.68 ± 0.40	0.64 ± 0.24	0.55 ± 0.20
		FIA	1.24 ± 0.34	0.66 ± 0.07	0.82 ± 0.18	0.74 ± 0.37
tph1	0.93 ± 0.16	CTRL	1.35 ± 0.47	0.95 ± 0.33	0.69 ± 0.20	0.86 ± 0.21
		FIA	1.36 ± 0.69	0.74 ± 0.21	0.92 ± 0.15	0.84 ± 0.32

Two-way ANOVA

Parameters	Time	Stimulus	Time × Stimulus	Time 4h	Time 24h	Time 48h	Time 72h	Stimulus CTRL	Stimulus FIA
gr1	0.003	-	-	b	ab	a	a	-	-
gr2	<0.001	-	-	c	bc	ab	a	-	-
htr1aβ	0.04	-	0.012	ab	a	ab	b	-	-
htr2a	0.01	-	-	b	ab	ab	a	-	-
htr2b	0.003	0.014	-	b	a	a	a	A	B
htr2c	-	-	0.001	-	-	-	-	-	-
ogfr1	-	-	<0.001	-	-	-	-	-	-
ogfr2	-	-	0.026	-	-	-	-	-	-
kor1	-	-	0.003	-	-	-	-	-	-
kor2	-	-	-	-	-	-	-	-	-
nopr	<0.001	-	-	b	a	a	a	-	-
muor	-	-	0.001	-	-	-	-	-	-
dor2	0.033	0.01	-	b	ab	ab	a	A	B
tph1	<0.001	-	-	b	a	a	a	-	-

One-way ANOVA

Parameters	i.p. injection
gr1	-
gr2	<0.001
htr1aβ	-
htr2a	-
htr2b	<0.001
htr2c	<0.001
ogfr1	-
ogfr2	-
kor1	-
kor2	-
nopr	<0.001
muor	<0.001
dor2	-
tph1	-

Values are presented as means ± SD (n=9). P-values from two-way and one-way ANOVA (p ≤0.05). If interaction was significant, Tukey post hoc test was used to identify differences in the experimental treatments. Different capital letters stand for significant differences between the two stimuli. Low case letters represent differences among sampling time. Different symbols indicate significant differences between i.p. injected and undisturbed fish.

Supplementary Table S2. Optic tectum expression of neuroendocrine genes in the European seabass i.p. injected with Freud's Incomplete Adjuvant (FIA) or Hank's Balanced Salt Solution (CTRL) and sampled at 4, 24, 48 or 72 h post injection.

Parameters	0h		4h	24h	48h	72h
gr1	1.04 ± 0.32	CTRL	1.32 ± 0.09	1.11 ± 0.18	1.20 ± 0.20	1.23 ± 0.13
		FIA	0.95 ± 0.12	0.92 ± 0.10	1.08 ± 0.11	1.22 ± 0.23
gr2	1.11 ± 0.48 #	CTRL	1.16 ± 0.24 B	1.06 ± 0.08	0.96 ± 0.14	0.96 ± 0.14
		FIA	0.68 ± 0.12 *Aa	1.01 ± 0.13 b	1.14 ± 0.03 b	1.11 ± 0.13 b
htr1αβ	1.03 ± 0.28	CTRL	1.12 ± 0.17	1.16 ± 0.20	1.11 ± 0.14	1.02 ± 0.31
		FIA	0.89 ± 0.15	1.07 ± 0.33	0.98 ± 0.21	0.91 ± 0.20
htr2a	0.97 ± 0.08	CTRL	1.20 ± 0.30	1.26 ± 0.28	1.19 ± 0.16	0.96 ± 0.27
		FIA	1.06 ± 0.14	1.00 ± 0.12	0.99 ± 0.21	0.97 ± 0.20
htr2b	1.09 ± 0.42	CTRL	1.35 ± 0.28	1.25 ± 0.46	0.97 ± 0.07	0.89 ± 0.07
		FIA	0.90 ± 0.21	1.25 ± 0.28	1.08 ± 0.35	1.09 ± 0.38
htr2c	1.03 ± 0.28	CTRL	1.33 ± 0.29	1.30 ± 0.28	1.34 ± 0.28	1.45 ± 0.34
		FIA	0.84 ± 0.28	1.19 ± 0.24	1.22 ± 0.14	1.45 ± 0.28
ogfr1	0.94 ± 0.16	CTRL	1.12 ± 0.15 B	1.10 ± 0.13	0.86 ± 0.15	1.01 ± 0.25
		FIA	0.71 ± 0.23 A	0.91 ± 0.15	0.89 ± 0.16	0.97 ± 0.17
ogfr2	1.03 ± 0.27 *	CTRL	0.91 ± 0.27 a	3.51 ± 0.63 #Bc	1.66 ± 0.63 #Bb	1.04 ± 0.24 ab
		FIA	0.69 ± 0.08	1.01 ± 0.27 A	0.97 ± 0.08 A	0.88 ± 0.26
kor1	0.90 ± 0.20	CTRL	1.07 ± 0.26	1.08 ± 0.09	1.32 ± 0.49	1.19 ± 0.25
		FIA	1.19 ± 0.28 ab	1.39 ± 0.56 b	1.04 ± 0.21 ab	0.78 ± 0.19 a
kor2	1.07 ± 0.44	CTRL	1.09 ± 0.25	1.15 ± 0.20	0.92 ± 0.12	0.88 ± 0.14
		FIA	0.78 ± 0.14	0.94 ± 0.13	0.83 ± 0.14	0.73 ± 0.22
nopr	1.01 ± 0.17	CTRL	1.29 ± 0.26 Bb	1.28 ± 0.23 Bb	0.78 ± 0.16 a	0.82 ± 0.12 a
		FIA	0.87 ± 0.14 A	0.88 ± 0.19 A	0.81 ± 0.17	0.80 ± 0.14
muor	0.93 ± 0.21 *	CTRL	1.31 ± 0.19 #	1.08 ± 0.19	1.02 ± 0.12	1.15 ± 0.24
		FIA	1.02 ± 0.23	0.96 ± 0.14	0.89 ± 0.07	1.01 ± 0.21
dor2	1.07 ± 0.40	CTRL	1.14 ± 0.31	1.46 ± 0.24	1.11 ± 0.17	1.32 ± 0.30
		FIA	0.83 ± 0.24	1.23 ± 0.14	1.12 ± 0.33	1.17 ± 0.16
tph1	1.20 ± 0.71	CTRL	1.61 ± 1.01	1.45 ± 0.57	1.66 ± 1.08	0.91 ± 0.49
		FIA	0.98 ± 0.72	0.94 ± 0.73	1.41 ± 0.40	0.88 ± 0.36

Two-way ANOVA									
				Time				Stimulus	
Parameters	Time	Stimulus	Time × Stimulus	4h	24h	48h	72h	CTRL	FIA
gr1	0.012	<0.001	-	ab	a	ab	b	B	A
gr2	-	-	<0.001	-	-	-	-	-	-
htr1aβ	-	0.032	-	-	-	-	-	B	A
htr2a	-	0.019	-	-	-	-	-	B	A
htr2b	-	-	-	-	-	-	-	-	-
htr2c	0.011	0.023	-	a	ab	ab	b	B	A
ogfr1	-	-	0.018	-	-	-	-	-	-
ogfr2	-	-	<0.001	-	-	-	-	-	-
kor1	-	-	0.046	-	-	-	-	-	-
kor2	0.01	<0.001	-	ab	b	ab	a	B	A
nopr	-	-	0.002	-	-	-	-	-	-
muor	0.046	0.002	-	b	ab	a	ab	B	A
dor2	0.003	0.014	-	a	b	ab	b	B	A
tph1	-	-	-	-	-	-	-	-	-

One-way ANOVA	
Parameters	i.p. injection
gr1	-
gr2	0.012
htr1aβ	-
htr2a	-
htr2b	-
htr2c	-
ogfr1	-
ogfr2	<0.001
kor1	-
kor2	-
nopr	<0.001
muor	0.01
dor2	-
tph1	-

Values are presented as means ± SD (n=9). P-values from two-way and one-way ANOVA (p ≤0.05). If interaction was significant, Tukey post hoc test was used to identify differences in the experimental treatments. Different capital letters stand for significant differences among sampling time. Low case letters represent differences between the two stimuli. Different symbols indicate significant differences between i.p. injected and undisturbed fish.

Supplementary Table S3. Hypothalamic expression of neuroendocrine genes in the European seabass i.p. injected with Freud's Incomplete Adjuvant (FIA) or Hank's Balanced Salt Solution (CTRL) and sampled at 4, 24, 48 or 72 h post injection.

Parameters	0h		4h	24h	48h	72h
gr1	1.04 ± 0.32 *	CTRL	0.88 ± 0.18 a	0.90 ± 0.14 a	1.21 ± 0.20 Aa	1.61 ± 0.15 #b
		FIA	1.04 ± 0.14 a	1.20 ± 0.10 a	1.68 ± 0.12 #Bb	1.57 ± 0.38 #b
gr2	1.07 ± 0.07	CTRL	0.94 ± 0.12 ab	0.79 ± 0.11 a	0.85 ± 0.17 Aab	1.13 ± 0.14 b
		FIA	0.85 ± 0.16 a	0.97 ± 0.06 ab	1.30 ± 0.14 Bb	1.27 ± 0.32 ab
crh	0.93 ± 0.27 *	CTRL	1.36 ± 0.26 ab	1.27 ± 0.32 a	1.33 ± 0.17 ab	1.78 ± 0.16 #b
		FIA	0.94 ± 0.11 a	0.95 ± 0.22 a	1.53 ± 0.51 #b	1.92 ± 0.42 #b
crhbp	1.10 ± 0.54	CTRL	1.09 ± 0.51	0.92 ± 0.37	0.94 ± 0.23	1.48 ± 0.30
		FIA	1.01 ± 0.42	0.54 ± 0.20	0.87 ± 0.19	1.44 ± 0.47
htr1aβ	1.03 ± 0.27	CTRL	1.08 ± 0.17 ab	0.84 ± 0.19 a	0.97 ± 0.22 Aa	1.30 ± 0.08 b
		FIA	0.96 ± 0.22 a	0.84 ± 0.20 a	1.31 ± 0.14 Bb	1.26 ± 0.07 b
htr2a	0.95 ± 0.10	CTRL	0.89 ± 0.14	0.71 ± 0.11	0.96 ± 0.26	0.82 ± 0.19
		FIA	0.85 ± 0.23	0.71 ± 0.25	1.04 ± 0.19	0.90 ± 0.14
htr2b	0.97 ± 0.26	CTRL	0.84 ± 0.41	0.89 ± 0.36	0.88 ± 0.33	1.20 ± 0.16
		FIA	0.80 ± 0.18	1.01 ± 0.40	1.13 ± 0.20	1.22 ± 0.22
htr2c	0.96 ± 0.10	CTRL	1.18 ± 0.26	0.82 ± 0.23	0.91 ± 0.11	1.15 ± 0.21
		FIA	0.95 ± 0.22	0.85 ± 0.17	1.19 ± 0.27	1.13 ± 0.32
ogfr1	0.96 ± 0.11 *	CTRL	1.06 ± 0.26 ab	0.83 ± 0.16 a	0.86 ± 0.14 Aa	1.36 ± 0.21 #b
		FIA	0.86 ± 0.18 a	0.97 ± 0.22 a	1.36 ± 0.15 #Bb	1.37 ± 0.25 #b
ogfr2	1.03 ± 0.25 *	CTRL	0.97 ± 0.21 a	3.30 ± 1.31 #Bb	1.92 ± 0.64 a	1.63 ± 0.38 a
		FIA	0.80 ± 0.09	1.10 ± 0.13 A	1.60 ± 0.03	1.62 ± 0.51
kor1	1.01 ± 0.18	CTRL	0.89 ± 0.16	0.75 ± 0.12	1.04 ± 0.18	1.15 ± 0.13
		FIA	0.83 ± 0.06	0.87 ± 0.30	1.32 ± 0.24	1.21 ± 0.30
kor2	0.92 ± 0.19 *	CTRL	1.10 ± 0.13 a	1.09 ± 0.60 a	1.37 ± 0.29 Aab	1.88 ± 0.22 #b
		FIA	1.15 ± 0.19 a	1.06 ± 0.45 a	2.06 ± 0.39 #Bb	1.57 ± 0.44 ab
nopr	1.02 ± 0.06	CTRL	0.93 ± 0.22	0.70 ± 0.12	0.92 ± 0.10	1.08 ± 0.33
		FIA	0.82 ± 0.28	0.70 ± 0.22	1.10 ± 0.19	0.92 ± 0.13
muor	1.38 ± 0.13 #	CTRL	1.13 ± 0.17	0.95 ± 0.33 *	1.07 ± 0.10	1.31 ± 0.18
		FIA	0.96 ± 0.06 *	0.98 ± 0.30 *	1.39 ± 0.23	1.44 ± 0.21

dor2	0.95 ± 0.47 #	CTRL	0.70 ± 0.23	0.67 ± 0.27	0.56 ± 0.15 A	0.89 ± 0.19
		FIA	0.68 ± 0.15 ab	0.46 ± 0.13 *a	1.09 ± 0.35 Bc	1.05 ± 0.20 bc
tph1	1.67 ± 0.42 #	CTRL	0.66 ± 1.14	0.58 ± 0.35 *	0.67 ± 0.56	0.69 ± 0.65
		FIA	0.48 ± 0.33 *	0.54 ± 0.53 *	1.20 ± 0.15	0.67 ± 0.64

Two-way ANOVA

Parameters	Time	Stimulus	Time × Stimulus	Time				Stimulus	
				4h	24h	48h	72h	CTRL	FIA
gr1	<0.001	<0.001	0.014	a	a	b	b	A	B
gr2	<0.001	0.001	0.006	a	a	ab	b	A	B
crh	-	-	0.022	-	-	-	-	-	-
crhbp	<0.001	-	-	a	a	a	b	-	-
htr1aβ	<0.001	-	0.01	ab	a	b	b	-	-
htr2a	0.006	-	-	ab	a	b	ab	-	-
htr2b	0.023	-	-	a	ab	ab	b	-	-
htr2c	0.012	-	-	ab	a	ab	b	-	-
ogfr1	-	-	<0.001	-	-	-	-	-	-
ogfr2	-	-	<0.001	-	-	-	-	-	-
kor1	<0.001	-	-	a	a	b	b	-	-
kor2	-	-	0.014	-	-	-	-	-	-
nopr	0.002	-	-	ab	a	b	b	-	-
muor	<0.001	-	-	ab	a	bc	c	-	-
dor2	-	-	<0.001	-	-	-	-	-	-
tph1	-	-	-	-	-	-	-	-	-

One-way ANOVA

Parameters	i.p. injection
gr1	<0.001
gr2	-
crh	<0.001
crhbp	-
htr1aβ	-
htr2a	-
htr2b	-
htr2c	-
ogfr1	<0.001
ogfr2	<0.001
kor1	-
kor2	<0.001
nopr	-
muor	<0.001
dor2	<0.001
tph1	0.02

Values are presented as means ± SD (n=9). P-values from two-way and one-way ANOVA ($p \leq 0.05$). If interaction was significant, Tukey post hoc test was used to identify differences in the experimental treatments. Different capital letters stand for significant differences among sampling time. Low case letters represent differences between the two stimuli. Different symbols indicate significant differences between i.p. injected and undisturbed fish.

Supplementary Table S4. Pituitary gland expression of neuroendocrine genes in the European seabass i.p. injected with Freud's Incomplete Adjuvant (FIA) or Hank's Balanced Salt Solution (CTRL) and sampled at 4, 24, 48 or 72 h post injection.

Parameters	0h		4h	24h	48h	72h
gr1	1.11 ± 0.67	CTRL	1.70 ± 0.30 b	1.11 ± 0.14 a	1.13 ± 0.15 a	1.10 ± 0.04 a
		FIA	1.61 ± 0.31 b	0.89 ± 0.08 a	1.34 ± 0.18 b	1.02 ± 0.19 a
pomc	1.03 ± 0.37	CTRL	1.30 ± 0.63	1.21 ± 0.16	1.39 ± 0.28	1.55 ± 0.29
		FIA	1.31 ± 0.25 ab	0.98 ± 0.25 a	1.50 ± 0.30 b	1.09 ± 0.28 ab
htr1aβ	1.00 ± 0.14 #	CTRL	0.29 ± 0.07 *b	0.22 ± 0.02 *ab	0.11 ± 0.01 *a	0.14 ± 0.06 *ab
		FIA	0.34 ± 0.05 *b	0.13 ± 0.06 *a	0.16 ± 0.08 *a	0.15 ± 0.07 *a
htr2a	1.00 ± 0.07 #	CTRL	0.41 ± 0.11 *	0.31 ± 0.08 *	0.23 ± 0.02 *	0.21 ± 0.13 *
		FIA	0.52 ± 0.12 *	0.21 ± 0.06 *	0.16 ± 0.04 *	0.16 ± 0.05 *
htr2b	1.00 ± 0.04	CTRL	0.92 ± 0.77	1.08 ± 0.15	0.85 ± 0.04	0.88 ± 0.40
		FIA	1.35 ± 0.41	0.89 ± 0.24	0.67 ± 0.17	1.01 ± 0.31
htr2c	1.03 ± 0.32 #	CTRL	0.78 ± 0.13	0.54 ± 0.14	0.81 ± 0.24	0.68 ± 0.28
		FIA	0.54 ± 0.21	0.36 ± 0.14 *	0.69 ± 0.28	0.55 ± 0.23
muor	1.00 ± 0.11	CTRL	1.40 ± 1.69	1.00 ± 0.40	0.64 ± 0.26	0.63 ± 0.38
		FIA	1.16 ± 0.35	0.55 ± 0.20	0.77 ± 0.22	0.49 ± 0.16
kor1	1.01 ± 0.24	CTRL	0.55 ± 0.62	0.57 ± 0.12	0.50 ± 0.21	0.64 ± 0.37
		FIA	0.77 ± 0.37	0.66 ± 0.18	0.79 ± 0.17	0.67 ± 0.30
dor2	1.13 ± 0.74 #	CTRL	0.64 ± 0.02	0.33 ± 0.09 *	0.31 ± 0.06 *	0.85 ± 0.22
		FIA	0.53 ± 0.07	0.23 ± 0.09 *	0.55 ± 0.34	0.47 ± 0.50

Two-way ANOVA									
				Time				Stimulus	
Parameters	Time	Stimulus	Time × Stimulus	4h	24h	48h	72h	CTRL	FIA
gr1	<0.001	-	0.013	c	a	b	a	-	-
pomc	0.013	-	0.053	ab	a	b	ab	-	-
htr1a	<0.001	-	0.032	b	a	a	a	-	-
htr2a	<0.001	-	-	b	a	a	a	-	-
htr2b	-	-	-	-	-	-	-	-	-
htr2c	0.011	0.02	-	ab	a	b	ab	B	A
muor	0.01	-	-	b	ab	a	a	-	-
kor1	-	-	-	-	-	-	-	-	-
dor2	0.004	-	-	ab	a	ab	b	-	-

One-way ANOVA	
Parameters	i.p. injection
gr1	-
pomc	-
htr1a	<0.001
htr2a	<0.001
htr2b	-
htr2c	0.007
kor1	-
muor	-
dor2	0.004

Values are presented as means ± SD (n=9). P-values from two-way and one-way ANOVA ($p \leq 0.05$). If interaction was significant, Tukey post hoc test was used to identify differences in the experimental treatments. Different capital letters stand for significant differences among sampling time. Low case letters represent differences between the two stimuli. Different symbols indicate significant differences between i.p. injected and undisturbed fish.
